# Supplementary material for: Mating-Type Locus Organization and Mating-Type Chromosome Differentiation in the Bipolar Edible Button Mushroom Agaricus bisporus
Source: Genes (Basel). 2021 Jul 16;12(7):1079. doi: 10.3390/genes12071079 (PMC8305134; doi:10.3390/genes12071079)
Supplement: Supplementary file 1 [file genes-12-01079-s001.zip › genes-1248252-supplementary.pdf]

Supplementary material

Mating-type locus organization and mating-type chromosomes in the bipolar edible button mushroom *Agaricus bisporus*

Marie Foulongne-Oriol[1], Ozgur Taskent[2], Ursula Kües[3], Anton S. M. Sonnenberg[4], Arend van Peer[4] and Tatiana Giraud[2]

[1] INRAE, MycSA, Mycologie et Sécurité des Aliments, 33882 Villenave d'Ornon, France

[2] Ecologie Systématique Evolution, Bâtiment 360, CNRS, AgroParisTech, Université Paris-Saclay, 91400 Orsay, France

[3] Molecular Wood Biotechnology and Technical Mycology, Büsgen-Institute and Goettingen Center for Molecular Biosciences (GZMB), University of Goettingen, Büsgenweg 2, 37077, Goettingen, Germany

[4] Plant Breeding, Wageningen University and Research, Droevendaalsesteeg 1, 6708 PB Wageningen, The Netherlands

A

```
a1-1  MTVSSIRK--------RLLQAEESFLAASNER--FMEDFEQTWNTLAVDIVKATSANQMSPEDKSLADIV  60
a1-2  MTISSIRK--------RLLHAEESFLAAKNDH--LMEDFEYAWTSLAVDIMKATSANQMTQEDIALADAV  60
b1-1  MSTRSSRKTVKQSLCRRFSDAESAFLDALDVGGGAMTAFEEEWTHLRDDFEAACIQGDVDPETASYARLV  70

a1-1  SSRITSLCKTFIDLTYPCDDISLSSEQELERILEEHESGNHGSQSRSSEETLHPAPALYIKQAYSWLMQN 130
a1-2  SSRITSLCKTFVEFTNACDDISLSFEQDLERILEEHDHGNHCSNHYSSEEMPRPAPAPYIKQAYSWLVQN 130
b1-1  TYRIAEISSSFLKLEATCKQLTDSLMADLASIPELRQS----QVLDTPQETISSR--RDVSLAAEWLSKN 134
                                                  ------------------------

a1-1  LSNPYPPKEVREAIARKAGSDPKHVENWFGDVRKRMGWNGIRKRHFSNKRHLTVAAATAFFGGQSSELLT 200
a1-2  LSNPYPPKEVREAIARKAGSDPKHVENWFGDARKRMGWNGIRKRHFSNKRRSTVAAATAFFGGQSSELLT 200
b1-1  FFNPYPSSTVRDRISHQSNWNRKDVDAWFTEARRRIGWNDIRKQYFQNKRAGAVERATEFFNG-SPTCFD 203
      -----------------------------------------

a1-1  DLVRQDFAAMSADAEDMYSKKFMESKLARHIAGVTGSPRKKPSQSAS-VENETSSSYNHP---------- 259
a1-2  DPVRQDFATMSADAEDMYSKKFMESKLARHIAGVTESPRKKRSQSAS-VEGGSSSSFNYS---------- 259
b1-1  AVLSQAFIDMESRVQDLYVEPYKPSKLAVVLG------KGHSDEAAFNMQKGFSTPFKSPRTLSPSRRSL 267

a1-1  ------LFHPSLPHTQCNA-RKRFRSLDSTDEVLDINNFQKRPRFSYSGDSSPAASHSPLPSTSPQSPVP 322
a1-2  ------LFHPSLPQTQCNP-RKRFRSLDLTDDVLDK---KKRPRLSYSGDSSPAALHSPLPTTSPQSPVP 319
b1-1  TSSSRSASPIDSSVSICQTNRKRPRS-DKTDQYHSQHP-RKKLR------------------EEEPTTIM 317
                           NLS                NLS

a1-1  HAIPATFQMNRQTSEFE---DHLPPSHPSADSRSV-SLQSVLELNRTSCNPSPSSSTLPSDVSDEPITSQ 388
a1-2  HAIPATFQMNCQTSEFE---DHLSPRHPSADSRPA-SLQSVQDLNRTSCNASPSSYTLPFDVSDEPITSQ 385
b1-1  LQAQPTCSSVSQSSLEEVSVDAIT-KGSSAVARPARSPRLSPFLDPQRKQLHPEALDLPNNAFGGSRTIR 386

a1-1  YIPNEATKRSDSVSMASFQQSP-TLHSSALSLLHNTLPSSLLSRKENTQFPTEFQAPVTYSEPVVTQGFA 457
a1-2  HIPNEDTKRSDLVSMASLRQSS-ILHSPALSLPRNILPSSLPSRKENTQFPTEFQMPVPCSESVVTQGFD 454
b1-1  SQTTSALGHTQSNACTPMAPDLGSINPTSLSNFRPSASSTIMPDLQVDFVHDISHAEI--SELSYTFPGP 454

a1-1  LAPSASDITHD---------TLFSGSTANDRFLAISSSDFAPRDLSSAQDVNATSWPATIANTQT----- 513
a1-2  LAPLASDVTHE---------TLFSGSTVNDRFLSTSHLDFASRSPSSVQDVNVTSWPGTIANTQI----- 510
b1-1  -------LTHDQPTLMQPVDQPWS-NSATSAHLPTYDLSMSTLLETASQAMPPNGNTDVVPSSDLFNNCS 516

a1-1  --DGQFFGYLDPLPTETTFTAQVDALPDFADVDLTFGNLFDLDASAAFPPSDPDFDT---ILPANRAENL 578
a1-2  --DGQFYGYLDSLPTEPNFTAQVDALPDFADVDLSFGNLFDLDASAAFPPSDPDFDS---MLPVNRAENL 575
b1-1  FYDDQLFELL-----RPPFGRGAPAVPPMQNFQG-LNTEVDISLAAPY----LDFESCTSQIPQPQIDDI 576

a1-1  ATNDLFTLTP----AVESSVSKFLQLEQLKKHRNRIQEYINTLEIQIATTLAA    627
a1-2  ATNDLLTLTT----AAENSVSKFLQLEQLKKHRNRIQDYINTLEIQIATTLAA    624
b1-1  LADFIHNFSAPPVDDAEK-LKRVAHVEMLKREKEHLQERLQSLYVSLPLVINKRLT 631
```

B

```
a2-1  MPAFRISDECSQRMNGVAS-VLSVLRKALCTKTPLPHSATLPSLSNIQLPQAPSIATVLKTHCVGGQSLAE  69
```

```
b2-2    MSPKIQETCQAILDSLRVPSFNISRPALQFGGLSNSSRNNWTLPPLQFPPIPSPAAQLVQLGCAFEESQ  69
b2-1                                               MPLILDPTP---ASIPHQRISDALSK  23

a2-1   ELTRTYEQHVSDLRKCTEAPIQGLLQHYRDN----------VISSDILEKQIARYISLYESRIKHWYNLL 129
b2-2   EAANIFASRVKDLQSTYERHYEKLFRRLSKYSSNTELRNLAKHINRVLDARYSRNITSWQEAILQAASIR 139
b2-1   SLSQSYQSKVNHVREVNFQKPRQVQYRLRQAPGESEQ--TPQIATDVISGAYMIQLKTWESHTLKKIARR  91

a2-1   SNEIKRGKS------EFNTERIPLLMTYFKHNAYPSIDDRNWLARETGNTPQQIGIWFQNRRRRSRKAGV 193
b2-2   VQERLKQASSTDERPVFKPEWTPFLKKYFAQNAYPTHQDKVILATKTGMTVKQIYDWFQNHRTREKRKGI 209
b2-1   LRK-NRGIEPTTCKPSFKYEYVPLLNSYFKRNAYPSTSDRASLAKKTNMSSRQIEVWFQNHRNRAHKNGQ 160
                ----------------------------------------------------------

a2-1   ELKRAGPDEISPNLFADDPEPYSPQPGTRPRVSSPEVT-TVEEVPLSCSSSVDATLRSHDHLRTHTDFTN 262
b2-2   PLKRPDIEGSPEFSSEDSPTSRVTTPEDDDMPLPKKRR-VATDEPLALSPVPSTSTQDFDFFCLPPDAVW 278
b2-1   ILNRVKDDQLPSDALPDEPPERSSTPPKNSHSSRKKCLSHSPSSSLQAPASPLSPTQCQSPPSIFSATCV 230

a2-1   FELPP-YAFPTTYNHPATPSCWSRPPETPFPEPNWDRK-------------PSSHRKNRHARSTATSVDD 318
b2-2   PRSYSEKDMKAFWECPVEPNLGSFTWPRELQPDSRSRK-------------RKALDSNIDEQIAESFASK 335
b2-1   QTISSTSKSSSPLDPPPLPYAFPPAFPRACDDDTFAIKGKDPWPFPAPQWIRLPAQTNVRKRKRTISIDE 300

a2-1   LSLMLAVKLRLFCNHPSSSAVADTPWHAT-LYTHTSPSPLPALVRSKQPYIITIGPTSQSIRSSLKRKRS 387
b2-2   LHFRSSLCKRTSLECPEETSDTFEPWSYHRLYPKRHPASVNYNQLTEPSKCAQPARPTELFPKYRRTLNS 405
b2-1   FAVEFAQHLSLCRHLGSEPSASKKKRSSWRDATHVYPCRGRHPALDLGQYQQKASLESSHHSPRHHSRPS 370

a2-1   EINEEQTCTKRFRSFSASSCSSFIGGDFYSSDDSSCTSSSPGPQTPPSAHLSLPPTPRFVDWSKFSIPTV 457
b2-2   SPSSRRRASSSSSHSTCSISSSTSPPSSIASSPPSMIRCSSWSSLDTLFTPGSSPRRPLQSLGISGLKGF 475
b2-1   ISSRRSSHTCRMPSGSESSVSTVSSPSSFDSTPLTSLDASPLLLPQNLDKAYASESSLSVQLQSLMLPGF 440

a2-1   PQAYGSC          464
b2-2   EVYAGPFVGLNMSISS 491
b2-1   LQYAGSSVKFGTAVS  455
```

Supplementary Figure S1 Alignment of **A.** *HD1* and **B.** *HD2* proteins from *Agaricus bisporus* var. *bisporus* H97 and *A. bisporus* var. *burnettii* JB137-S8. The positions of the homeodomains are marked by a dashed line. NLS marks motifs of alike structure of the bipartite nuclear localisation signals in *Coprinopsis cinerea* (Spit *et al.*, 1998) and *Laccaria bicolor* (Niculita-Hirzel *et al.*, 2008).

A

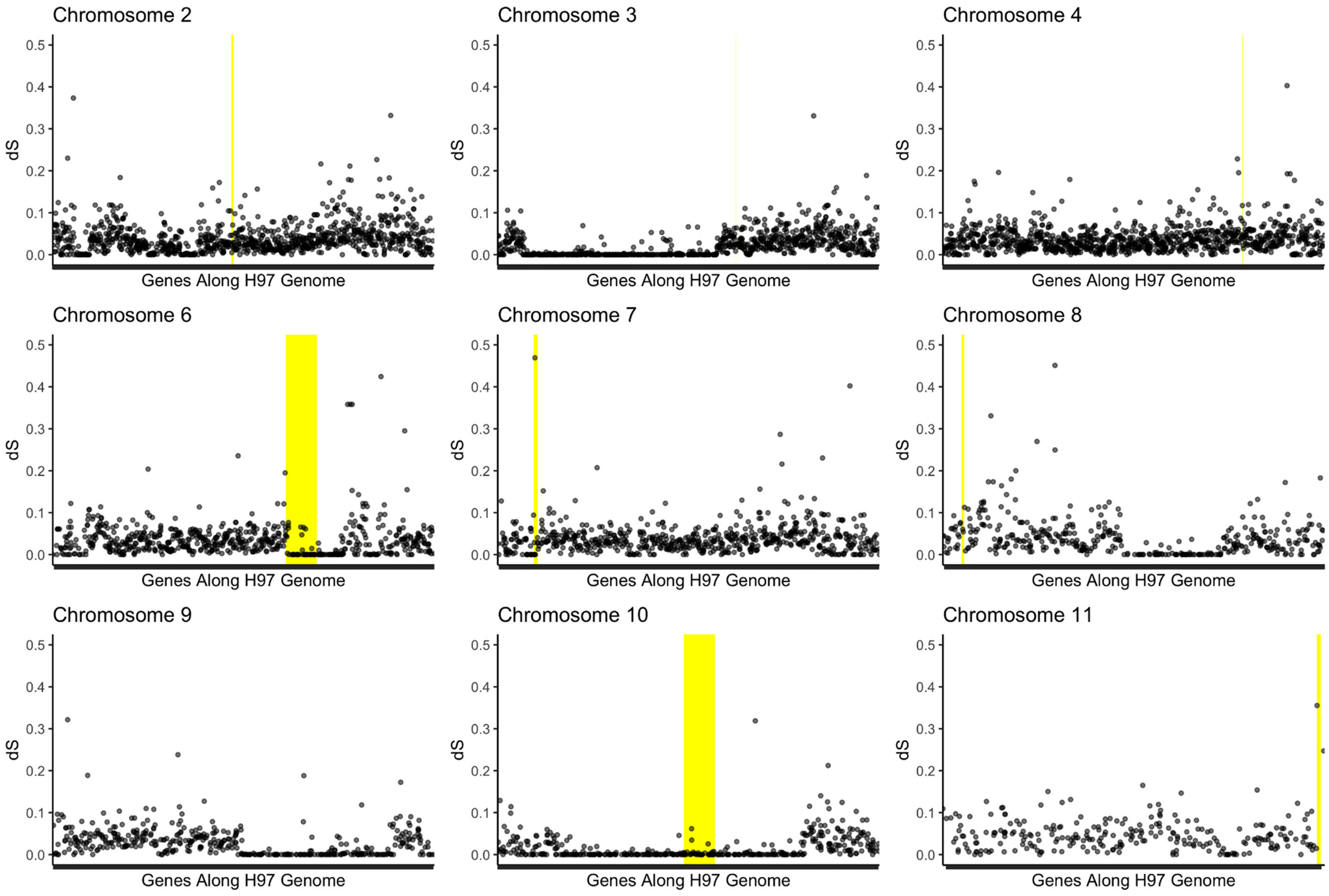

Chromosome 2
Chromosome 3
Chromosome 4
Chromosome 6
Chromosome 7
Chromosome 8
Chromosome 9
Chromosome 10
Chromosome 11
dS
0.5
0.4
0.3
0.2
0.1
0.0
Genes Along H97 Genome

B

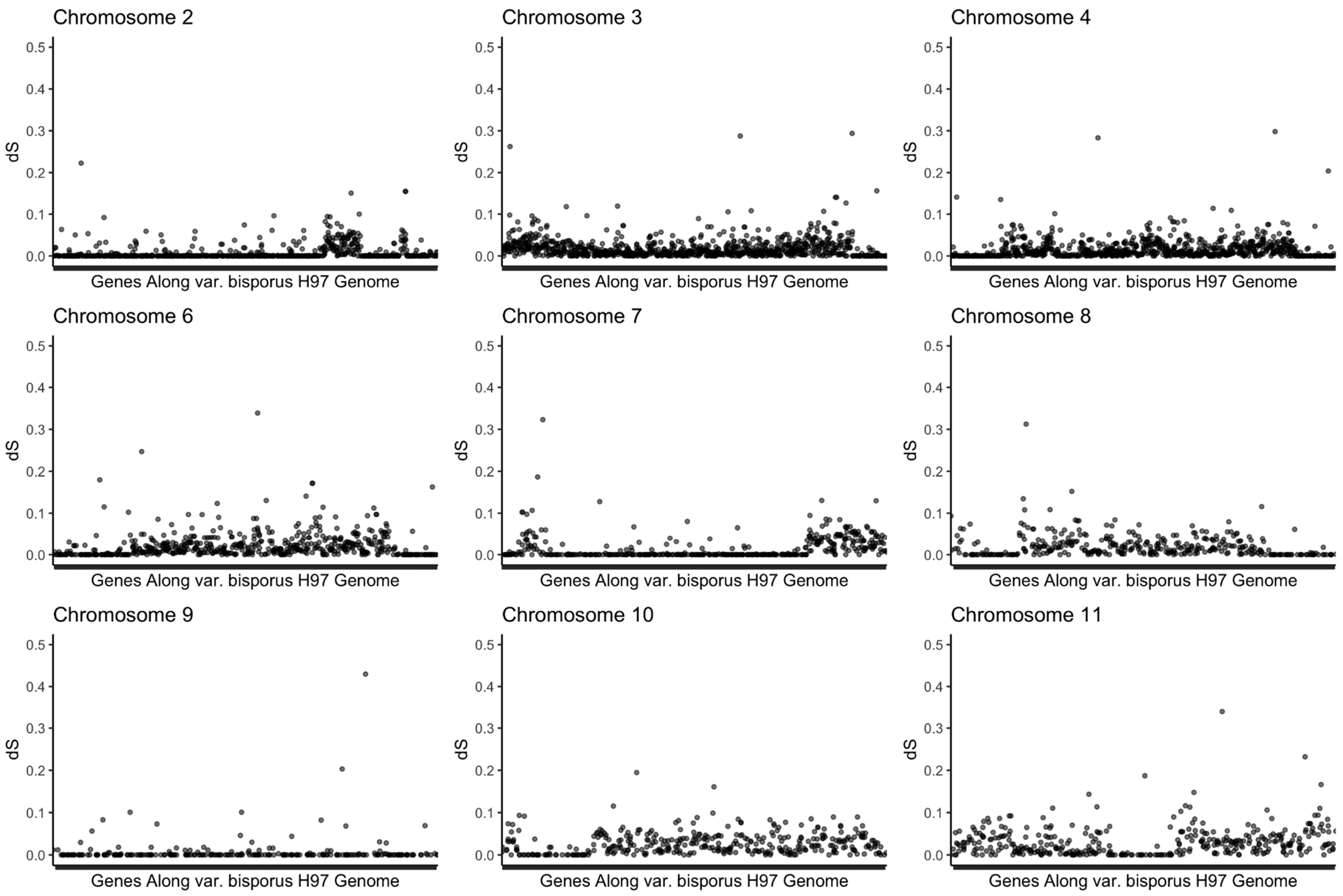

Supplementary Figure S2. Synonymous substitution rates between the homokaryotic genomes of *Agaricus bisporus* var. *bisporus* H97 and H39 (A) and between *A. bisporus* var. *burnettii* H119p1 and H119p4 (B). Y-axis shows the synonymous divergence levels between the alleles found in the homokaryotic genomes of H97 and H39 of alternative mating types, issued from the U1 cultivar. X-axis shows the gene order along the H97 genome. Yellow highlights on the background shows the putative centromeric regions. The other chromosomes are shown in Figure 8.

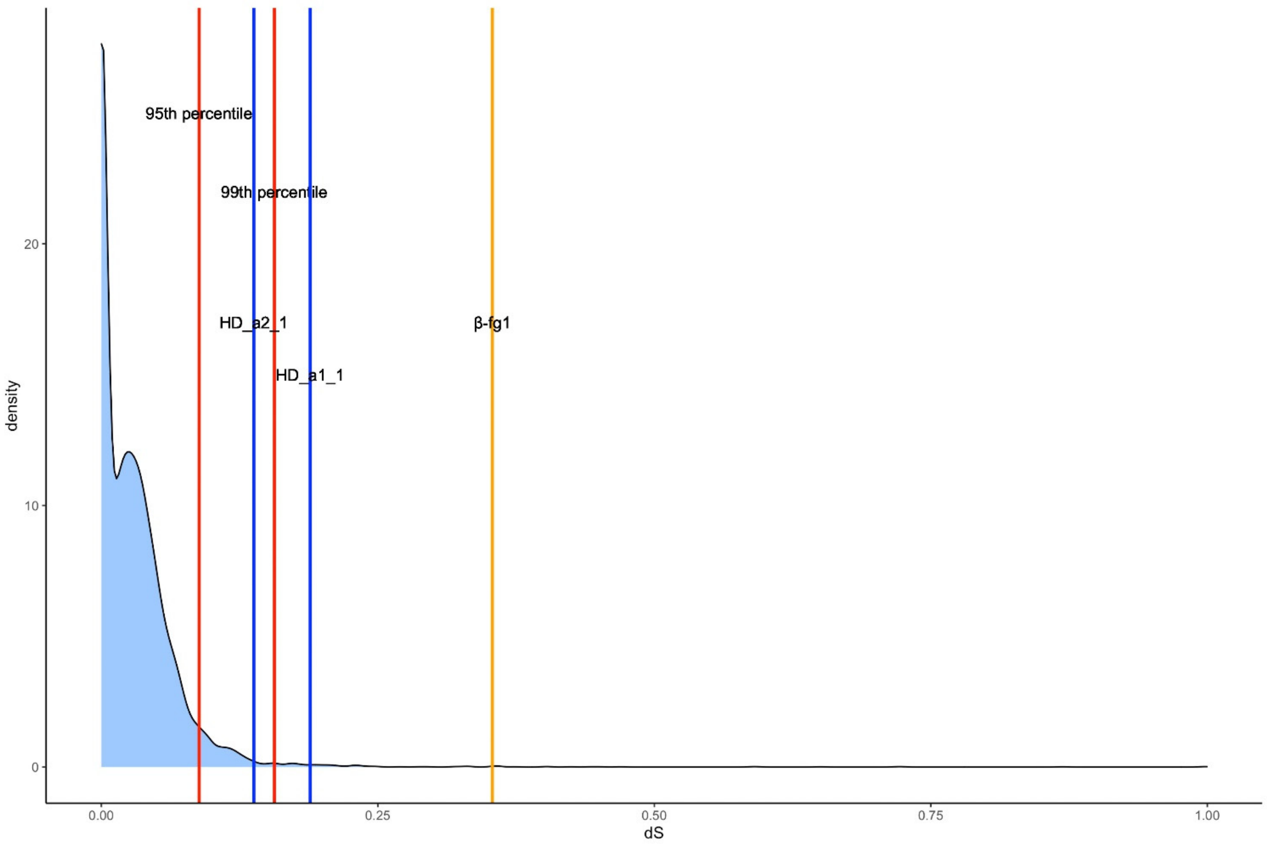

Supplementary Figure S3. Distribution of the rate of synonymous substitutions (dS) between orthologous gene pairs found in the constitutive homokaryon genomes of H97 and H39 of *Agaricus bisporus* var. *bisporus* Hurst U1. Density plot shows the relative frequencies of genes with different dS values. The 95th and 99th percentile values of the distribution are highlighted with vertical red lines. The dS values for the two *HD* genes and *β-fg1* gene are shown with blue and orange vertical lines, respectively.

Supplementary Table S1 : Gene comparison between *Agaricus bisporus* var *bisporus* H97 v3.0 and *Agaricus bisporus* var *burnettii* JB137-S8: genomic position in the two strains, DNA identity and protein identity

Agaricus bisporus var *bisporus* H97 v3.0		*Agaricus bisporus* var *burnettii* JB137-S8		DNA identity (%)		Protein identity (%)
Gene ID/name	Position (start to stop codon)	Gene ID/name	Position (start to stop codon)	Genomic DNA	Coding sequence	
239372/*mip1*	scaffold_1:897420-899760 (+)	67360/*mip1*	scaffold_1:2040314-2042755 (-)	98	98	99
113833/*a1-1*	scaffold_1:895062-897100 (+)	124448/*a1-2*	scaffold_1:2043142-2045173 (-)	88	89	82
239365/*a2-1*	scaffold_1:893233-894863 (-)	144391/remnant of *a2-2*	scaffold_1:2045374-2045559 (+)	77	-*	-
-	-	144392/footprint of a *mip1* gene	scaffold_1:2045600-2045806 (-)	(77 to *mip1*)	-*	-
-	-	144356/*b1-1*	scaffold_1:2080256-2082318 (-)	-	-	-
239364/*b2-2*	scaffold_1:890959-892499 (-)	124456/*b2-1*	scaffold_1:2082520-2084040 (+)	-	-	26
1181595/*β-fg2*	scaffold_1:887425-889480 (+)	144375/*β-fg2*	scaffold_1:2084717-2086770 (-)	98	99	98
1137415/*β-fg1*	scaffold_1:883864-884895 (+)	81839/*β-fg1*	scaffold_1:2087937-2088968 (-)	94	96	98
239575/*phl1*	scaffold_1:2918372-2918506 (+)	144336/*phl1*	scaffold_1:59978-60141 (-)	99	99	98
239576/*phl2*	scaffold_1:3231293-3231622 (+)	144361/*phl2*	scaffold_14:29026-29157 (+)	100	100	100
239578/*phl3*	scaffold_1:3236723-3236857 (-)	144359/*phl3*	scaffold_14:34681-34815 (-)	99	99	98
239573/*phl4*	scaffold_5:200825-200999 (-)	86158/*phl4*	scaffold_9:175788-175616 (-)	97	97	96
239579/*ste3.1*	scaffold_5:2047209-2048830 (-)	121275/*ste3.1*	scaffold_7:898975-900596 (-)	98	99	97
239590/*ste3.2*	scaffold_13:276835-278479 (+)	144367/*ste3.2*	scaffold_17:267571-269202 (+)	98	98	98
239584/*ste3.3*	scaffold_13:392785-394687 (+)	144363/*ste3.3*	scaffold_17:379554-381458 (+)	98	98	98
195352/*ste3.4*	scaffold_12:1236632-1238485 (-)	123419/*ste3.4*	scaffold_38:95442-97029 (+)	96	97	98

*contains frameshift(s) and stop codon(s)

Supplementary Table S2: Microsynteny analysis performed by tblastn searches on JGI page http://genome.jgi-psf.org/Copci1/Copci1.home.html (settings: default) with genes of the regions with *HD* mating type genes on the chromosomes 1 of *Agaricus bisporus* var. *bisporus* H97 (scaffold 1) compared to the genome of *Coprinopsis cinerea* Okayama 7 (no hit: -)

***Agaricus bisporus* var. *bisporus* H97**		***Coprinopsis cinerea* Okayama 7**	
**Gene**	**Position on chromosome I**	**Gene**	**Position on chromosome I**
189405 (*pab1*)	932956-935538 (-)	CC1G_01849T0 (*pab1*)	2699003-2701362 (-)
1165947	931452-932725 (-)	-	-
1120398 (*up1*)	930119-931431(+)	CC1G_01848T0	2697506-2698930 (+)
1165946 (*ubc12*)	928622-930005 (-)	CC1G_01847T0	2696166-2697285 (-)
1072572	928377-928937 (+)	CC1G_13707T0	2695644-2696187 (+)
1153365 (*up3*)	927132-928291 (-)	CC1G_01846T0	2694335-2695507 (-)
1095010 (*up4*)	924999-927074 (-)	CC1G_01845T0	2692021-2694125 (-)
1072437 (*up5*)	922378-924450 (-)	CC1G_13706T0	2689754-2691849 (-)
1181608 (*up6*)	919573-922217 (-)	CC1G_13705T0	2686968-2688467 (-)
1145681 (*glydh*)	915514-919100 (-)	CC1G_01843T0	2683012-2686558 (-)
1153361 (*up7*)	912956-915306 (+)	CC1G_01842T0	2680968-2682946 (+)
1031411 (*sec61*)	912405-912835 (-)	CC1G_01841T0	2680073-2680505 (-)
1095004	910403-912306 (+)	CC1G_01840T0	2678200-2679927 (+)
1103173 (*up8*)	906900-909825 (+)	CC1G_01839T0	2674086-2677215 (+)
1165935 (*up9*)	905234-906831 (+)	CC1G_01838T0	2672530-2673857 (+)

1137419 (*up10*)	904507-905196 (-)	CC1G_01837T0	2671654-2672349 (-)
1165933	902316-904458 (-)	-	-
1072561 (*up11*)	900667-902087 (-)	CC1G_01836T0	2669318-2671452 (-)
239372 (*mip1*)	897420-899938 (-)	CC1G_01835T0 (*mip1*)	2666417-2668995 (-)
113833 (*a1-1*)	895062-897100 (+)	CC1G_01834T0 (*a1-2*)	2666140-2664189 (+)
239365 (*a2-1*)	893233-894463 (-)	CC1G_01833T0 (*a2-2*)	2663799-2662251 (-)
-	-	CC1G_01832T0	2660867-2660048 (+)
-	-	CC1G_01831T0 (*b1-2*)	2655981-2653848 (+)
239364 (*b2-2*)	890959-892499 (-)	Modified CC1G_13704T0 (*b2-2*)	2653400-2651473 (-)
-	-	CC1G_01828T0 (*d1-1*)	2653400-2651473 (-)
1181595 (*β-fg2*)	887425-889480 (+)	-	-
1137415 (*β-fg1*)	883864-884895 (+)	CC1G_01827T0 (*β-fg*)	2646406-2647545 (+)
1181614	882720-883653 (+)	CC1G_01826T0	2645288-2646234 (+)
1094996 (*glgen*)	879120-882345 (+)	CC1G_01825T0	2641261-2644545 (+)
1094995	876725-878971 (-)	CC1G_01824T0	2638742-2641057 (-)
1103148 (*blac2*)	874323-876620 (+)	-	-
1160308 (*mad2*)	873029-874228 (-)	CC1G_01823T0	2637048-2637945 (-)
1165929 (*snx4*)	871257-873344 (+)	CC1G_01822T0	2634949-2636783 (+)

1031396 (*up12*)	868800-871138 (-)	CC1G_01821T0	2632303-2634830 (-)
1031395 (*rpb2*)	864573-868430 (+)	CC1G_13703T0 (two genes fused to one model)	2626696-2631959 (+)
1120344 (*up13*)	862870-864414 (+)		
1165927 (*fmnor1*)	860988-862678 (-)	CC1G_01818T0	2624916-2626437 (-)
1181620 (*qdr1*)	858946-860793 (+)	-	-
1103133 (*ndse*)	857528-858720 (+)	CC1G_01817T0	2623644-2624843 (+)
1153345 (*upa4*)	855520-857395 (+)	CC1G_01816T0	2621439-2623236 (+)
1024634	853801-855409 (-)	CC1G_01815T0	2619103-2621082 (-)
213701	850192-853206 (+)	CC1G_01814T0	2615859-2618928 (+)
1165924	849031-850133 (+)	CC1G_01813T0	2614502-2615625 (+)
1153341	845123-848866 (-)	CC1G_01812T0	2610634-2614222 (-)
1094983	840285-843205 (+)	CC1G_01811T0	607625-2610066 (+)
1024630	838934-839727 (+)	CC1G_01810T0	2606242-2607178 (+)
1120321 (*cwc25*)	837108-838693 (-)	CC1G_01809T0	2604610-2606051 (-)
1137404(*lim15/dmc1*)	836248-837091 (-)	CC1G_01808T0	2602748-2604371 (-)
1165918 (*RNAb30*)	833488-834784 (-)	-	-
1165917 (*hst3*)	830976-833064 (-)	-	-
61363 (*rp6*)	829899-830250 (+)	CC1G_01807T0	2601740-2602693 (+)

1094976	824356-828040 (-)	CC1G_01806T0	2597092-2600714 (-)
-	-	CC1G_01805T0	2595167-2596736 (+)
1153333	821738-823496 (+)	CC1G_01804T0	2592521-2594140 (+)
1072421	820710-821631 (+)	CC1G_13702T0	2591415-2592320 (+)
1120304	819167-820691 (+)	CC1G_01803T0	2589970-2591279 (+)
1145648	817392-818862 (-)	CC1G_01802T0	2588157-2589877 (-)
1094972	815356-817579 (+)	CC1G_01801T0	2586076-2588027 (+)
1044354	813756-814924 (+)	CC1G_01800T0	2584273-2585762 (+)
1120294	812245-813487 (-)	CC1G_01799T0	2582928-2584111 (-)
-	-	CC1G_01798T0	2581293-2582126 (+)
1072934	810638-812175 (+)	CC1G_01797T0	2578317-2579859 (+)
1165910	809176-810262 (-)	CC1G_01796T0	2576916-2577993 (-)
1103082	807160-808228 (-)	CC1G_01795T0	2575851-2576750 (-)
1165908	802076-804569 (-)	CC1G_01794T0	2572983-2575510 (-)
1153321	797672-802037 (+)	CC1G_13701T0	2568876-2572693 (-)
1153320	795950-797603 (-)	CC1G_01791T0	2567390-2568674 (-)
213648	794661-796492 (+)	CC1G_01790T0	2564978-2566678 (+)
1165904	793411-794430 (-)	CC1G_01789T0	2562478-2563426 (-)

		CC1G_01788T0	2561151-2562077 (-)
1120267	791196-793007 (-)	CC1G_01787T0	2559457-2561006 (-)
1165902	788993-790798 (-)	CC1G_01786T0	2557814-2558980 (-)
1103060	787718-789588 (+)	CC1G_01785T0	2556370-2557695 (+)
1165901 (*gpd*)	785731-787605 (-)	CC1G_01784T0	2554397-2556162 (-)
147399	783211-785503 (-)	CC1G_01783T0	2551954-2554178 (-)
1103054	780120-782052 (-)	-	-
1103058 (*ade8*)	776958-779907 (+)	CC1G_01782T0 (*ade8*)	2548109-2551563 (+)

References

Niculita-Hirzel H, Labbe J, Kohler A, le Tacon F, Martin F, Sanders IR, Kues U. 2008. Gene organization of the mating type regions in the ectomycorrhizal fungus *Laccaria bicolor* reveals distinct evolution between the two mating type loci. *New Phytologist* **180**(2): 329-342.

Spit A, Mellor EJC, Casselton LA. 1998. A role for heterodimerization in nuclear localization of a homeodomain protein. *Proc. Natl. Acad. Sci, USA* **95**: 6228-6233.
